# Decision‐Making and Alzheimer's Research (DMAR): A longitudinal DCE study examining supportive care preferences among older adults with dementia and their care partners

**DOI:** 10.1002/alz70858_098596

**Published:** 2025-12-25

**Authors:** Anne M Turner

**Affiliations:** ^1^ University of Washington, Seattle, WA, USA

## Abstract

**Background:**

Older adults with dementia are often left out of decision making about their care, particularly as the disease progresses. The Decision‐Making in Alzheimer's Research (DMAR) project is a 5‐year NIH funded project conducted through the University of Washington. The purpose is to design a choice‐based tool for persons with dementia (PWD) and their care partners (CP) to better understand preferences when making decisions about supportive care.

**Methods:**

Initial formative work included in‐depth interviews with PWD (*n* = 24), CP (*n* = 37) and providers/staff (*n* = 18) to understand factors affecting decisions when seeking more supportive care. Applying human centered design (HCD) we sought input from PWD and CP to develop an accessible Discrete Choice Experiment (DCE) instrument, using think‐aloud methods. We are conducting a 12‐month longitudinal study using our DCE instrument to compare the weighted preferences of PWD and CP and compare preferences over time.

**Results:**

Through a person‐centered approach we successfully enrolled and administered the DCE to 131 dyads (PWD and CP). We are analyzing the results of the baseline and 6‐month DCEs to compare weighted preferences of PWD and CP and identify changes in preferences over time.

**Conclusion:**

Through a person‐centered process we successfully developed and administered a DCE instrument to 131 dyads for our longitudinal dyad study. In addition to the longitudinal study results, we will share strategies for inclusive recruitment and the impact of the dementia‐centered design on data modeling and analysis. The use of new technologies to facilitate engagement of older adults with dementia will be explored.

